# Real-world data on the use of the Shingrix vaccine among patients with inflammatory arthritis and risk of cardiovascular events following herpes zoster

**DOI:** 10.1186/s13075-025-03565-0

**Published:** 2025-05-17

**Authors:** Jeffrey R. Curtis, Danielle M. Conrad, Whitney S. Krueger, Andrew P. Gara, Kevin L. Winthrop

**Affiliations:** 1 Division of Clinical Immunology and Rheumatology, University of Alabama at Birmingham, Birmingham, AL USA; 2 AbbVie Inc., North Chicago, IL USA; 3 Schools of Medicine and Public Health, Oregon Health and Science University, Portland, OR USA

**Keywords:** Arthritis, Cardiovascular disease, Herpes zoster, Inflammatory disease, Real-world studies, Vaccines

## Abstract

**Background:**

Risk of cardiovascular events may increase after herpes zoster; therefore, American College of Rheumatology guidelines strongly recommend vaccination against herpes zoster in patients aged ≥ 18 years with rheumatic and musculoskeletal diseases taking immunosuppressive medications. Here, we investigated the effectiveness of Shingrix among patients with inflammatory arthritis and estimated the post-herpes zoster risk of cardiovascular events.

**Methods:**

In this retrospective observational cohort study, data were obtained from the Optum™ Clinformatics™ Data Mart on patients aged ≥ 18 years with rheumatoid arthritis, psoriatic arthritis, or axial spondyloarthritis. The proportions of patients receiving any Shingrix dose, a second dose, and a second dose within 6, 9, and 12 months were calculated. Incidence of herpes zoster following inflammatory arthritis diagnosis was reported. Vaccine effectiveness was calculated as (1 – incidence rate ratio of herpes zoster) × 100. Relative risk of cardiovascular events was assessed independently in the 30-, 45-, 60-, and 90-day periods post-herpes zoster in a subgroup of patients who experienced cardiovascular events.

**Results:**

The final cohort included 132,672 patients with inflammatory arthritis. Mean age was 60.4 years, 71.9% were female, and 80.0% were diagnosed with rheumatoid arthritis. Overall, 28,690 (21.6%) patients received ≥ 1 Shingrix dose, of whom only 73.2% received a second dose. Of those receiving a second dose, 17,598 (83.8%) received it within the recommended 2–6 months after the first. Herpes zoster occurred in 4,342 (3.3%) patients, of which 360 cases occurred after Shingrix vaccination. The incidence rate (95% confidence interval) of herpes zoster per 1,000 person-years was 7.41 (6.64, 8.17) after any Shingrix vaccination vs. 14.76 (14.30, 15.22) without vaccination (crude vaccine effectiveness: 50%). The risk of venous thromboembolic events was elevated in the 60–90 days post-herpes zoster; no significantly increased risk was observed for any other cardiovascular events.

**Conclusions:**

This study showed that the effectiveness of Shingrix in patients with inflammatory arthritis on immunomodulatory treatment was 50%, and the risk of venous thromboembolic events was increased in the 60–90 days after herpes zoster, supporting the recommendation that adults with inflammatory arthritis should receive vaccination against herpes zoster to reduce the risk of such events.

**Supplementary Information:**

The online version contains supplementary material available at 10.1186/s13075-025-03565-0.

## Background

Herpes zoster, or shingles, is caused by reactivation of the varicella zoster virus (VZV) and is a relatively common infection among older adults [1]. The incidence of herpes zoster among the general population is 4–7 cases per 1,000 person-years (PY), with an estimated lifetime risk of 20–30% that increases with age [2]; most cases occur in adults over the age of 50 years [3]. Primary VZV infections usually occur in childhood and lead to varicella, or chickenpox, after which VZV remains dormant for long latency periods prior to potential reactivation as herpes zoster [1]. An important factor leading to VZV reactivation is immunosenescence; accordingly, herpes zoster risk increases in older adults and/or immunocompromised individuals, including those with autoimmune conditions or who are receiving immunosuppressant medications [1, 2].

The first vaccine for herpes zoster available in the US was Zostavax, a live attenuated VZV vaccine. Zostavax was first approved by the Food and Drug Administration (FDA) in 2006 for adults ≥ 60 years old, then expanded in 2011 to include adults ≥ 50 years old [4, 5]. However, as a live virus vaccine, few patients with immunosuppressive conditions, or patients receiving immunosuppressive treatments, received it [6]. More recently, Shingrix, a recombinant, adjuvanted VZV vaccine, was approved in the US in October 2017 for adults ≥ 50 years old [7]; the greater efficacy of Shingrix led to a preferential recommendation over Zostavax by the Center for Disease Control Advisory Committee on Immunization Practices (CDC ACIP) and resulted in Zostavax no longer being available for use in the US (since July 2020) [8]. The clinical trials of Shingrix conducted in relatively healthy individuals free of immunosuppressive conditions found that Shingrix is highly effective at preventing herpes zoster; results from those trials indicated that Shingrix is 97.2% effective among adults ≥ 50 years old [9]; > 89% in adults ≥ 70 years old [10]; and real-world effectiveness is estimated to be 79.2% in adults ≥ 50 years old [11].

Current American College of Rheumatology (ACR) 2022 guidelines state that, for patients aged ≥ 18 years with rheumatic and musculoskeletal diseases who are taking immunosuppressive medications, VZV vaccination is strongly recommended [12]. A post hoc analysis of clinical trial data among participants with at least one potential immune-mediated disease (most commonly psoriasis, spondyloarthropathy, and rheumatoid arthritis) estimated Shingrix efficacy at 90.5% [13]. However, patients who were immunosuppressed were not permitted to enter those trials, and thus, individuals receiving typical systemic treatments (e.g., biologic disease-modifying antirheumatic drugs [bDMARDs], Janus kinase [JAK] inhibitors, conventional synthetic disease-modifying antirheumatic drugs [csDMARDs], and higher-dose glucocorticoids) were excluded, severely limiting the generalizability of that post hoc result to the rheumatology patients seen in routine practice. Data on the real-world effectiveness of any herpes zoster vaccine among people with autoimmune conditions (including psoriatic arthritis and rheumatoid arthritis) are extremely limited. For Zostavax, effectiveness has been estimated at 33.0–39.0% [11]; for Shingrix, lower seroconversion rates (74%) were observed in patients with immune-mediated rheumatic diseases treated with JAK inhibitors compared with healthy controls (96%), emphasizing the need for tailored vaccination strategies in such patients to optimize vaccine efficacy [14]. There is growing evidence that Shingrix effectively prevents herpes zoster and related complications in a range of at-risk immunocompromised patient populations aged ≥ 18 years [15], and post-marketing data in Medicare patients aged > 65 years showed vaccine effectiveness of 64.1% and 70.5% in immunocompromised and immunocompetent patients, respectively [16]. However, there are no large-scale studies of the recombinant, adjuvanted VZV vaccine in patients with autoimmune and inflammatory rheumatic diseases receiving typical immunomodulatory treatments.

Risk of cardiovascular events (myocardial infarction, stroke, other major adverse cardiovascular events [MACE], and venous thromboembolic events [VTEs]) may be increased after herpes zoster [17–24]. Particularly for complicated herpes zoster (e.g., with cranial nerve involvement), the risk of stroke has been estimated to be ~ 30–50% higher during the first 6 months after herpes zoster, declining after 1 year [17–21, 23, 24]. The risk of myocardial infarction is also elevated [19, 21, 22, 24]. It is unknown whether Shingrix vaccination, and thus a reduction in the risk of herpes zoster, may also reduce the risk of cardiovascular events, including among patients with rheumatologic conditions [17]. The current study utilized a US administrative claims database to describe the characteristics of patients with inflammatory arthritis receiving Shingrix, to determine the effectiveness of Shingrix among this group of patients. We also estimated the risk of cardiovascular events following herpes zoster as a first step to consider whether Shingrix might reduce that risk.

## Methods

### Study overview

This was a retrospective observational cohort study. Data were obtained from the Optum™ Clinformatics™ Data Mart (Optum CDM), which is a database containing administrative health claims from a large US national managed care company. Claims were verified, adjudicated, adjusted, and de-identified. The patient population included in the dataset comprised commercial health plan members and Medicare Advantage members across all 50 states and Puerto Rico. Patients were only included in the database if they had both medical and prescription drug coverage. Data pertaining to inpatient confinements (i.e., hospitalizations), member eligibility, and outpatient laboratory tests (if processed by a company under contract with the managed care organization) were included. Data were accessed and analyzed through the Aetion® Substantiate Platform, a secure web-based software package designed for real-world data analytics.

The objectives of this study were: 1) to describe the population of patients with inflammatory arthritis receiving Shingrix; 2) to estimate the effectiveness of Shingrix in preventing herpes zoster among patients with inflammatory arthritis; and 3) to estimate the risk of cardiovascular events (myocardial infarction, stroke, combined stroke or myocardial infarction [two-part MACE; hereafter MACE], and VTE) after herpes zoster among patients with inflammatory arthritis.

### Study population

The primary cohort consisted of patients with rheumatoid arthritis, psoriatic arthritis, or axial spondyloarthritis who met the following specific cohort entry requirements: 1) aged ≥ 18 years on cohort entry date; 2) cohort entry date (date of meeting rheumatologic diagnostic criteria) between October 20, 2017 (FDA Shingrix approval date) and last available data cut-off point (March 31, 2023); 3) ≥ 365 days of continuous enrollment prior to cohort entry date (receipt of a rheumatologic medication within 365 days of diagnosis), with a ≤ 45-day gap in enrollment; and 4) ≥ 1 day of continuous enrollment after the cohort entry date. Patients who met the following criteria within the 365 days prior to cohort entry were excluded: 1) evidence of any hematologic or solid malignancy (excluding non-melanoma skin cancer) requiring chemotherapy or radiation treatment; 2) prior solid organ transplant; 3) human immunodeficiency virus/acquired immune deficiency syndrome; 4) receipt of antiviral therapy (acyclovir, valacyclovir, famciclovir); or 5) missing information on age or sex.

For objective 3, a nested self-controlled case series (SCCS) study design, common in vaccine research, that has the advantage of minimizing confounding by allowing patients to serve as their own control [25], was used to study a subgroup of patients who experienced cardiovascular events. The following washout periods were included: 1) no diagnosis of herpes zoster or postherpetic neuralgia in the 365 days prior to cohort entry; 2) for myocardial infarction and stroke analyses, patients had to be free from myocardial infarction and stroke, respectively (or both for MACE analyses), in the 90 days prior to cohort entry; 3) for VTE analyses, patients had to be free from VTEs in the 180 days prior to cohort entry.

### Exposures

The inflammatory arthritis conditions of interest were rheumatoid arthritis, psoriatic arthritis, and axial spondyloarthritis (including both ankylosing spondylitis and non-radiographic axial spondyloarthritis), defined as ≥ 2 claims for any diagnostic International Classification of Disease codes (identified by a rheumatologist; Supplementary Table 1) occurring ≥ 7 days and ≤ 365 days apart, and use of licensed rheumatologic medications for these conditions within 365 days after the initial diagnosis claim.

Shingrix is designed to be given in two doses, 2–6 months apart. The proportion of patients receiving any dose, the proportion of patients receiving a second dose, and the proportions of patients receiving a second dose within 6, 9, and 12 months were calculated. Rheumatologic medications allowed included csDMARDs, bDMARDs, and JAK inhibitors.

### Outcomes

Baseline patient characteristics, comorbidities, medication, and healthcare utilization prior to cohort entry, and the incidence of herpes zoster following inflammatory arthritis diagnosis, were reported. For objective 1, the proportion of patients with inflammatory arthritis who received one dose of Shingrix vaccine, the proportion of patients who received a second dose of Shingrix, and the proportions of patients who received a second dose within 2–6 months (the recommended time interval), 2–9 months, and 2–12 months, were reported. For objective 2, the incidence of herpes zoster following inflammatory arthritis diagnosis was reported, comparing vaccinated with unvaccinated patients. To increase specificity, herpes zoster events were defined as a diagnosis claim with receipt of antiviral medication within ± 7 days. Vaccine effectiveness was calculated as (1 – incidence rate ratio [IRR] of herpes zoster) × 100. For objective 3, the relative risk of cardiovascular events (myocardial infarction, stroke, MACE, and VTE [all required inpatient diagnosis with hospitalization for ≥ 1 day] based on validated algorithms with a positive predictive value of 90% for MACE [26] and 91% for VTE [27, 28]) were assessed independently in the 30-, 45-, 60-, and 90-day time periods following herpes zoster diagnosis, in a subgroup of patients who experienced cardiovascular events, consistent with the SCCS design.

### Covariates

Baseline patient characteristics were assessed using the last value observed on the cohort entry date, and included age, sex, race, and US/Puerto Rico geographic region. Cardiovascular risk factors were assessed using all available data prior to and including the cohort entry date. Baseline medications use, healthcare utilization, and herpes zoster risk factors were all assessed using the latest available data within 365 days prior to and including cohort entry. A list of covariates can be found in Supplementary Table 2.

### Statistical analyses

For baseline patient characteristics, continuous variables were reported as mean ± standard deviation, median (interquartile range), or range (min–max), and categorical variables were reported as proportions. For each event of interest following inflammatory arthritis diagnosis, crude incidence rates (IRs) per 1,000 PY (95% confidence intervals [CIs]) were reported. For all analyses, patients were followed up until end of available data, disenrollment, death, or the outcome of interest occurred, after which they were censored. For objective 1, analyses were descriptive only. For objective 2, analyses consisted of estimates of the incidence of herpes zoster following inflammatory arthritis diagnosis and vaccination. Poisson regression models were used to estimate the IR and 95% CI of herpes zoster per 1,000 PY. IRs were stratified by Shingrix vaccination status. Analytic follow-up time began on the cohort entry date for non-vaccinated individuals; for vaccinated individuals, analytic person-time began after the first dose of vaccine.

For objective 3, analyses used an SCCS design [25] in a subgroup of patients who experienced cardiovascular events, and consisted of estimates of the relative risk (IRRs [95% CI]) of myocardial infarction, stroke, MACE, and VTE following herpes zoster using a conditional Poisson regression model. The SCCS model compared the incidence of cardiovascular events across different risk periods after herpes zoster within individuals. SCCS implicitly controls for non-time-varying confounders by comparing patients to themselves in different risk intervals, seeking to answer questions related to the timing of a hypothesized exposure (i.e., recent herpes zoster). Analytic person-time began on October 20, 2017. Person-time prior to herpes zoster was defined as the control period, and person-time after herpes zoster was defined as the risk period. Shingrix vaccination status was treated as a time-varying covariate in the model. Additional adjustment for time-varying covariates including age and medication use (rheumatologic medications, glucocorticoids) were added to the models. Age was categorized into < 50, 50 to < 70, and ≥ 70 years. Rheumatologic medication use was treated as a time-varying hierarchical exposure, with patients being categorized according to their highest level of exposure if taking more than one class of drug: csDMARDs > bDMARDs > JAK inhibitors. Concomitant glucocorticoid use was allowed. Glucocorticoid use was measured as a categorical time-varying exposure. Categories were defined by dose: 0 mg/day prednisone equivalent, 0–7.5 mg/day prednisone equivalent, and > 7.5 mg/day prednisone equivalent. A list of rheumatologic medications can be found in Supplementary Table 3.

## Results

### Patients and cohort baseline characteristics

**Fig. 1:**
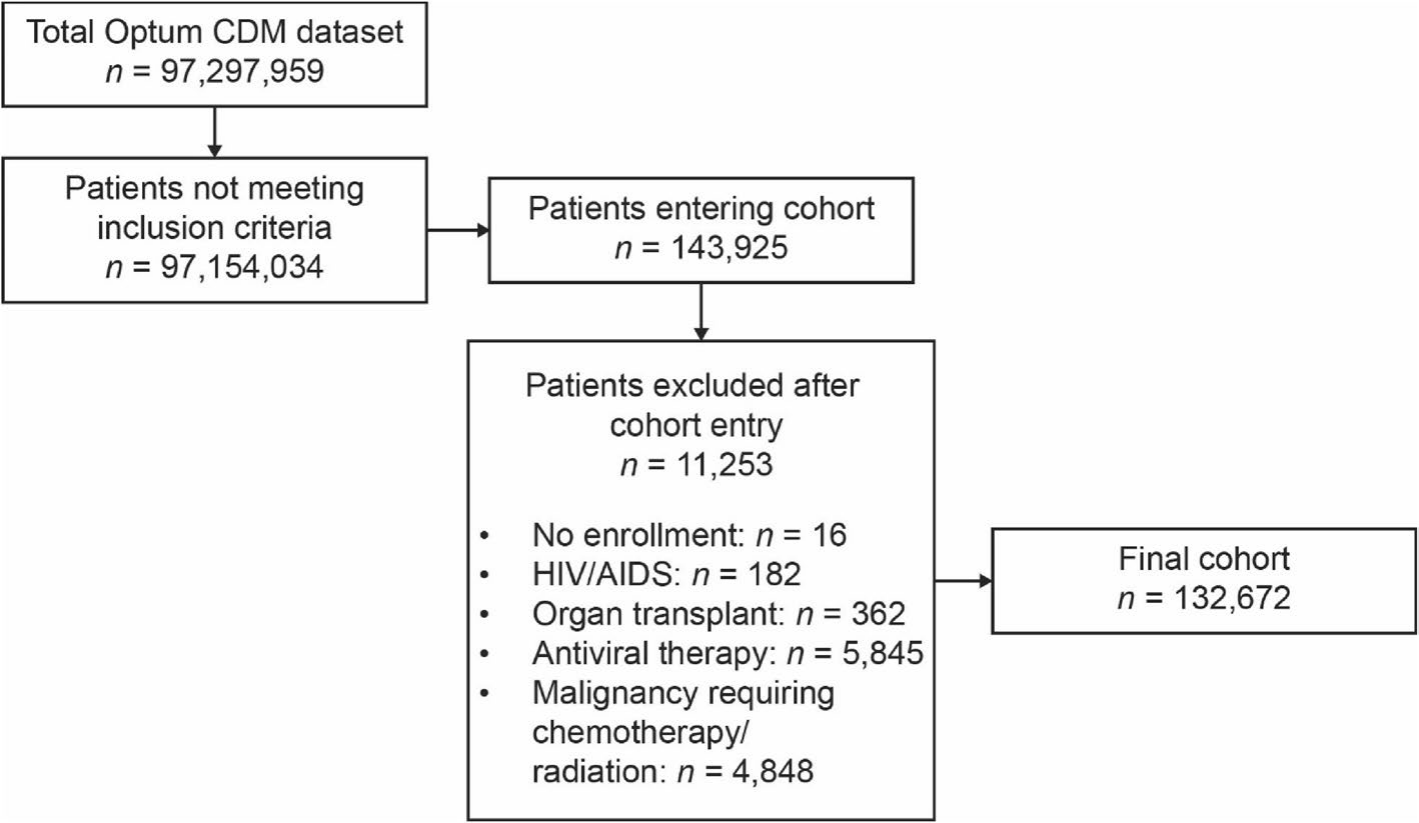
Patient flowchart. *AIDS* acquired immune deficiency syndrome, *HIV* human immunodeficiency virus, *Optum CDM* Optum™ Clinformatics™ Data Mart

Of 97,297,959 patients in the Optum CDM dataset, 132,672 patients with inflammatory arthritis met the criteria for inclusion in the final cohort (Fig. 1). The mean age was 60.4 years, 71.9% were female, and the majority (80.0%) were diagnosed with rheumatoid arthritis (Table 1). In total, 79.6%, 39.4%, and 7.5% of patients were receiving csDMARDs, bDMARDs, or JAK inhibitors, respectively, at any time on or after cohort entry (Table 2). Non-steroidal anti-inflammatory drugs and glucocorticoids were prescribed for 51.1% and 75.3% of patients, respectively.

**Table 1:** Baseline demographics and patient characteristics stratified by inflammatory arthritis diagnosis

	**Total population**	**RA**	**PsA**	**axSpA**
***N* (%)**	132,672	106,098 (80.0)	21,871 (16.5)	6,643 (5.0)
**Age, mean (SD)**	60.36 (14.04)	61.77 (13.64)	56.26 (13.66)	49.36 (14.58)
**Age groups, *n* (%)**				
< 50	28,541 (21.5)	19,205 (18.1)	6,696 (30.6)	3,370 (50.7)
50 to < 70	67,295 (50.7)	54,155 (51.0)	11,447 (52.3)	2,680 (40.3)
≥ 70	36,836 (27.8)	32,738 (30.9)	3,728 (17.0)	593 (8.9)
**Gender, *n* (%)**				
Male	37,217 (28.1)	25,048 (23.6)	9,316 (42.6)	3,534 (53.2)
Female	95,455 (71.9)	81,050 (76.4)	12,555 (57.4)	3,109 (46.8)
**Race, *n* (%)**				
White	92,848 (70.0)	72,611 (68.4)	16,806 (76.8)	4,844 (72.9)
Asian	3,709 (2.8)	2,873 (2.7)	622 (2.8)	259 (3.9)
Black	14,363 (10.8)	12,724 (12.0)	1,282 (5.9)	491 (7.4)
Hispanic	15,109 (11.4)	12,597 (11.9)	2,073 (9.5)	679 (10.2)
Missing	6,643 (5.0)	5,293 (5.0)	1,088 (5.0)	370 (5.6)
**Geographic region, *n* (%)**				
Northeast	14,554 (11.0)	11,425 (10.8)	2,711 (12.4)	626 (9.4)
South	64,744 (48.8)	52,301 (49.3)	10,429 (47.7)	3,203 (48.2)
Midwest	31,935 (24.1)	25,299 (23.8)	5,470 (25.0)	1,496 (22.5)
West	21,355 (16.1)	17,007 (16.0)	3,250 (14.9)	1,311 (19.7)
Missing/unknown/Puerto Rico	84 (0.1)	66 (0.1)	11 (0.1)	7 (0.1)
**Inflammatory arthritis diagnosis, *n* (%)**				
RA	106,098 (80.0)	106,098 (100.0)	987 (4.5)	736 (11.1)
PsA	21,871 (16.5)	987 (0.9)	21,871 (100.0)	278 (4.2)
axSpA	6,643 (5.0)	736 (0.7)	278 (1.3)	6,643 (100.0)
**Comorbidities, *n* (%)**				
Alcohol use	3,498 (2.6)	2,642 (2.5)	702 (3.2)	198 (3.0)
Asthma	24,762 (18.7)	20,305 (19.1)	3,731 (17.1)	1,094 (16.5)
Arrhythmias	9,004 (6.8)	7,677 (7.2)	1,126 (5.1)	297 (4.5)
Chronic kidney disease	15,770 (11.9)	13,481 (12.7)	1,985 (9.1)	461 (6.9)
Chronic liver disease	20,755 (15.6)	16,329 (15.4)	3,796 (17.4)	997 (15.0)
Chronic obstructive pulmonary disease	30,105 (22.7)	25,484 (24.0)	3,990 (18.2)	994 (15.0)
Congestive heart failure	10,915 (8.2)	9,506 (9.0)	1,229 (5.6)	282 (4.2)
Depression	36,104 (27.2)	28,906 (27.2)	6,007 (27.5)	1,763 (26.5)
Diabetes	35,648 (26.9)	29,100 (27.4)	5,869 (26.8)	1,162 (17.5)
Drug abuse	7,768 (5.9)	6,260 (5.9)	1,242 (5.7)	402 (6.1)
Dyslipidemia	79,252 (59.7)	64,728 (61.0)	12,629 (57.7)	2,962 (44.6)
Herpes simplex virus	4,322 (3.3)	3,468 (3.3)	713 (3.3)	219 (3.3)
Herpes zoster (plus antivirals)	5,396 (4.1)	4,588 (4.3)	669 (3.1)	197 (3.0)
Hypertension	84,241 (63.5)	69,207 (65.2)	12,994 (59.4)	3,147 (47.4)
Ischemic heart disease	26,531 (20.0)	22,316 (21.0)	3,698 (16.9)	845 (12.7)
Family history of ischemic heart disease	10,941 (8.2)	8,772 (8.3)	1,808 (8.3)	516 (7.8)
Myocardial infarction	2,140 (1.6)	1,826 (1.7)	266 (1.2)	77 (1.2)
Stroke	1,878 (1.4)	1,643 (1.5)	201 (0.9)	51 (0.8)
MACE	4,149 (3.1)	3,574 (3.4)	494 (2.3)	131 (2.0)
VTE	1,616 (1.2)	1,402 (1.3)	163 (0.7)	66 (1.0)
Obesity	49,469 (37.3)	38,986 (36.7)	9,114 (41.7)	2,181 (32.8)
Peripheral vascular disease	21,641 (16.3)	18,679 (17.6)	2,631 (12.0)	547 (8.2)
Smoking (ever)	41,325 (31.1)	33,923 (32.0)	6,269 (28.7)	1,678 (25.3)
Current smoking (past year)	6,930 (5.2)	5,747 (5.4)	1,008 (4.6)	287 (4.3)
**Medications, *n* (%)**				
Opioid use	78,933 (59.5)	63,549 (59.9)	12,675 (58.0)	3,956 (59.6)
Zostavax vaccination	6,124 (4.6)	5,311 (5.0)	729 (3.3)	140 (2.1)
Shingrix vaccination	8,518 (6.4)	6,911 (6.5)	1,367 (6.3)	356 (5.4)
**Healthcare utilization**				
ER visit (past year), *n* (%)	33,327 (25.1)	27,729 (26.1)	4,652 (21.3)	1,410 (21.2)
ER visit (ever), *n* (%)	59,276 (44.7)	48,727 (45.9)	8,715 (39.8)	2,648 (39.9)
Outpatient rheumatologist visit (past year), *n* (SD)	2.87 (2.09)	2.91 (2.13)	2.71 (1.93)	2.68 (2.06)
Outpatient rheumatologist visit (ever), *n* (SD)	7.72 (10.58)	7.96 (10.86)	6.80 (9.43)	6.67 (9.41)
Outpatient visit (past year), *n* (SD)	10.97 (7.78)	11.12 (7.80)	10.48 (7.60)	10.21 (7.83)
Outpatient visit (ever), *n* (SD)	38.27 (43.90)	38.99 (44.84)	35.76 (39.84)	34.80 (40.21)

Inflammatory arthritis diagnoses are not mutually exclusive. Covariate status was assessed across all available data before cohort entry unless otherwise stated

*axSpA* axial spondyloarthritis, *ER* emergency room, *MACE* major adverse cardiovascular events, *PsA* psoriatic arthritis, *RA* rheumatoid arthritis, *SD* standard deviation, *VTE* venous thromboembolic event

**Table 2:** Medication use (any time on or after cohort entry)

	**Total population**	**RA**	**PsA**	**axSpA**
***N***	132,672	106,098	21,871	6,653
**csDMARD, *n* (%)**	105,661 (79.6)	89,316 (84.2)	14,977 (68.5)	2,744 (41.2)
Apremilast	3,518 (2.7)	470 (0.4)	3,150 (14.4)	73 (1.1)
Chloroquine	67 (0.1)	61 (0.1)	5 (< 0.1)	1 (< 0.1)
Hydroxychloroquine	44,350 (33.4)	42,346 (39.9)	1,759 (8.0)	676 (10.2)
Leflunomide	20,692 (15.6)	18,425 (17.4)	2,161 (9.9)	348 (5.2)
Methotrexate	61,346 (46.2)	51,456 (48.5)	9,393 (42.9)	1,260 (18.9)
Sulfasalazine	16,346 (12.3)	13,049 (12.3)	2,415 (11.0)	1,084 (16.3)
**bDMARD, *n* (%)**	52,294 (39.4)	36,694 (34.6)	11,555 (52.8)	5,087 (76.5)
Abatacept	2,671 (2.0)	5,233 (4.9)	460 (2.1)	34 (0.5)
Adalimumab	22,283 (16.8)	14,154 (13.3)	5,963 (27.3)	2,691 (40.4)
Anakinra	143 (0.1)	141 (0.1)	0 (0.0)	3 (< 0.1)
Certolizumab pegol	6,352 (4.8)	4,199 (4.0)	1,567 (7.2)	751 (11.3)
Etanercept	11,111 (8.4)	7,842 (7.4)	2,516 (11.5)	928 (13.9)
Golimumab	6,425 (4.8)	4,351 (4.1)	1,469 (6.7)	779 (11.7)
Infliximab	6,198 (4.7)	3,985 (3.8)	1,553 (7.1)	831 (12.5)
Rituximab	2,835 (2.1)	2,762 (2.6)	70 (0.3)	19 (0.3)
Sarilumab	615 (0.5)	605 (0.6)	9 (< 0.1)	5 (0.1)
Tocilizumab	2,333 (1.8)	2,318 (2.2)	20 (0.1)	12 (0.2)
**JAK inhibitors, *n* (%)**	10,006 (7.5)	8,755 (8.3)	1,194 (5.5)	212 (3.2)
Baricitinib	452 (0.3)	440 (0.4)	13 (0.1)	4 (0.1)
Tofacitinib	7,170 (5.4)	6,150 (5.8)	1,006 (4.6)	116 (1.7)
Upadacitinib	3,381 (2.5)	3,092 (2.9)	240 (1.1)	110 (1.7)
**NSAIDs, *n* (%)**	67,858 (51.1)	53,753 (50.7)	11,453 (52.4)	3,765 (56.6)
**Glucocorticoids (prednisone equivalent)**^**a**^**, *n* (%)**				
None	32,775 (24.7)	20,003 (18.9)	10,174 (46.5)	3,279 (49.3)
≤ 7.5 mg/day	33,967 (25.6)	30,951 (29.2)	2,683 (12.3)	671 (10.1)
> 7.5 mg/day	65,930 (49.7)	55,144 (52.0)	9,014 (41.2)	2,703 (40.6)

^a^Highest daily dose at any time on or after cohort entry. Inflammatory arthritis diagnoses are not mutually exclusive. Covariate status was assessed across all available data before cohort entry unless otherwise stated

*axSpA* axial spondyloarthritis, *bDMARD* biologic disease-modifying antirheumatic drug, *csDMARD* conventional synthetic disease-modifying antirheumatic drug, *JAK* Janus kinase, *NSAID* non-steroidal anti-inflammatory drug, *PsA* psoriatic arthritis, *RA* rheumatoid arthritis

A total of 28,690 (21.6%) patients were vaccinated with at least one dose of Shingrix, of whom only 73.2% received a second dose (Table 3). Of those receiving a second dose, it was received within the recommended timeframe of 2–6 months after the first dose by 17,598 (83.8%) patients. A total of 19,226 (91.5%) and 19,757 (94.0%) patients had received the second dose within 2–9 or 2–12 months, respectively. Most patients (*n* =  24,475; 85.3%) had ≥ 180 days of enrollment after Shingrix vaccination (Table 3), lessening concern that lack of observing a second dose was an artifact of loss of coverage and observability in the data. When stratified by Shingrix use, the proportion of patients vaccinated increased with age and was greater in patients diagnosed with rheumatoid arthritis than with psoriatic arthritis or axial spondyloarthritis (Supplementary Table 4). Comorbidities including dyslipidemia, hypertension, and ischemic heart disease were more common among vaccinated patients.

**Table 3:** Outcomes (stratified by inflammatory arthritis diagnosis)

	**Total population**	**RA**	**PsA**	**axSpA**
***N***	132,672	106,098	21,871	6,643
**Any Shingrix dose, *n* (%)**	28,690 (21.6)	23,482 (22.1)	4,498 (20.6)	1,052 (15.8)
**Any second Shingrix dose**	21,012 (73.2)	17,265 (73.5)	3,252 (72.3)	743 (70.6)
Second Shingrix dose 2–6 months after first dose	17,598 (83.8)	14,443 (83.7)	2,751 (84.6)	613 (82.5)
Second Shingrix dose 2–9 months after first dose	19,226 (91.5)	15,781 (91.4)	2,998 (92.2)	675 (90.8)
Second Shingrix dose 2–12 months after first dose	19,757 (94.0)	16,219 (93.9)	3,071 (94.4)	699 (94.1)
** ≥ 180 days’ enrollment after Shingrix vaccination, *n* (%)**	24,475 (85.3)	20,090 (85.6)	3,796 (84.3)	885 (84.1)
**Herpes zoster, *n* (%)**	4,342 (3.3)	3,684 (3.5)	559 (2.6)	140 (2.1)
IR per 1,000 PY (95% CI)	13.64 (13.23, 14.04)	14.27 (13.81, 14.73)	11.13 (10.21, 12.05)	9.88 (8.24, 11.51)
**Myocardial infarction, *n* (%)**	3,361 (2.5)	2,926 (2.8)	400 (1.8)	63 (0.9)
IR per 1,000 PY (95% CI)	10.43 (10.08, 10.78)	11.20 (10.79, 11.60)	7.88 (7.11, 8.66)	4.39 (3.31, 5.48)
**Stroke, *n* (%)**	2,979 (2.2)	2,617 (2.5)	324 (1.5)	50 (0.8)
IR per 1,000 PY (95% CI)	9.23 (8.90, 9.56)	10.00 (9.62, 10.39)	6.37 (4.58, 7.07)	3.48 (2.51, 4.44)
**MACE, *n* (%)**	6,227 (4.7)	5,429 (5.1)	720 (3.3)	119 (1.8)
IR per 1,000 PY (95% CI)	19.56 (19.08, 20.05)	21.05 (20.49, 21.61)	14.32 (13.27, 15.36)	8.34 (6.84, 9.84)
**VTE, *n* (%)**	1,975 (1.5)	1,754 (1.7)	189 (0.9)	47 (0.7)
IR per 1,000 PY (95% CI)	6.10 (5.83, 6.37)	6.68 (6.36, 6.99)	3.70 (3.18, 4.23)	3.27 (2.33, 4.20)

Shingrix counts were assessed across all available data. Other outcomes (herpes zoster, stroke, myocardial infarction, MACE, and VTE) could occur only after cohort entry (i.e., after inflammatory arthritis diagnosis) due to software restrictions in calculation of person-time at risk

*axSpA* axial spondyloarthritis, *CI* confidence interval, *IR* incidence rate, *MACE* major adverse cardiovascular events, *PsA* psoriatic arthritis, *PY* person-years, *RA* rheumatoid arthritis, *VTE* venous thromboembolic event

### Outcomes

**Fig. 2:**
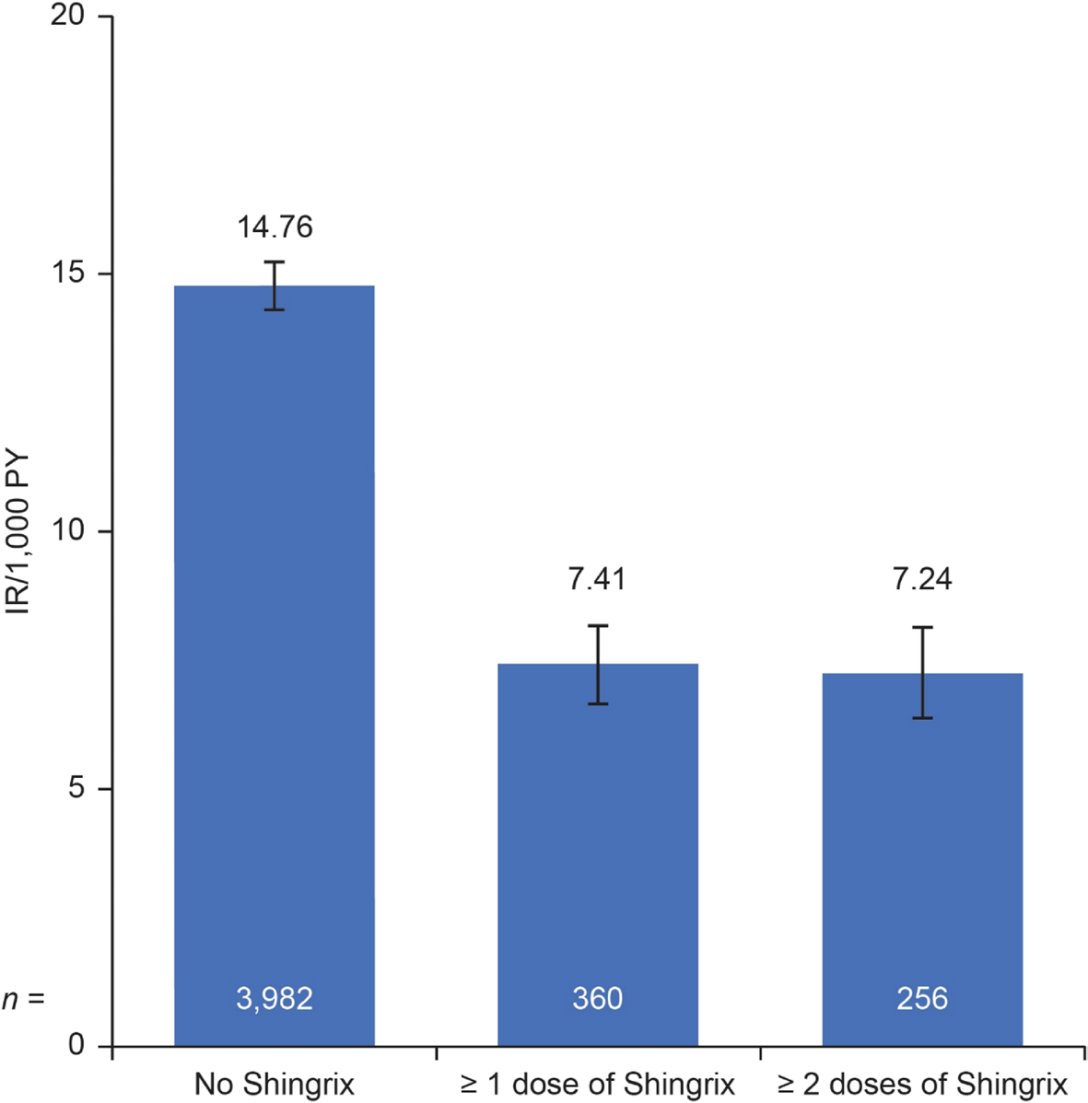
IR of herpes zoster by Shingrix vaccination status. Error bars represent 95% CI. The crude incidence rate ratio of herpes zoster comparing “Any Shingrix” to “No Shingrix” was 0.5 (95% CI: 0.44, 0.55). *CI* confidence interval, *IR* incidence rate, *PY* person-years

Herpes zoster occurred in 4,342 (3.3%) patients, of which 360 cases occurred after Shingrix vaccination (Table 3; Fig. 2). The IR of herpes zoster was 11.35 (95% CI: 10.50, 12.21) in the < 50 years age group vs. 14.99 (95% CI: 14.23, 15.75) in the ≥ 70 years age group (Supplementary Fig. 1). The IR of herpes zoster after any Shingrix vaccination was 7.41 (95% CI: 6.64, 8.17) per 1,000 PY compared with 14.76 (95% CI: 14.30, 15.22) per 1,000 PY without vaccination, yielding a crude vaccine effectiveness of 50% (95% CI: 44, 55) (Fig. 2).

**Fig. 3:**
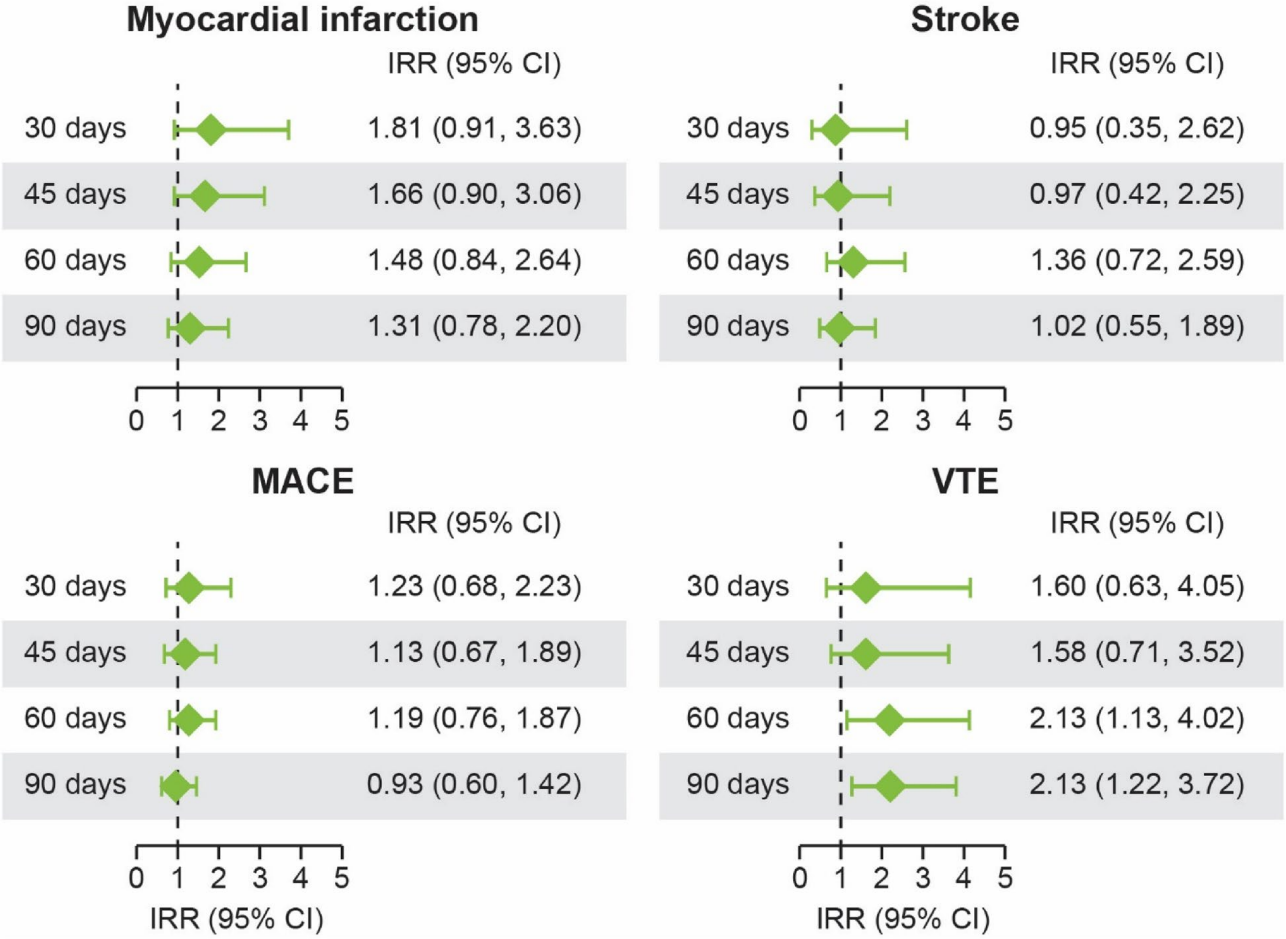
Summary of key data from models (i.e., relative incidence rates at 30, 45, 60, and 90 days after herpes zoster). *CI* confidence interval, *IRR* incidence rate ratio, *MACE* major adverse cardiovascular events, *VTE* venous thromboembolic event

When outcomes were stratified by age, an increased risk of myocardial infarction (< 50 years, IR = 1.78 [95% CI: 1.45, 2.12]; 50 to < 70 years, IR = 8.43 [95% CI: 7.98, 8.87]; ≥ 70 years, IR = 18.94 [95% CI: 18.09, 19.79]), stroke (< 50 years, IR = 1.78 (1.45, 2.11); 50 to < 70 years, IR = 6.57 (6.17, 6.97); ≥ 70 years, IR = 18.10 [95% CI: 17.26, 18.93]), MACE (< 50 years, IR = 3.72 [95% CI: 3.24, 4.20], 50 to < 70 years, IR = 14.92 [95% CI: 14.32, 15.52]; ≥ 70 years, IR = 37.07 [95% CI: 35.86, 38,28]) and VTE (< 50 years, IR = 1.85 [95% CI: 1.51, 2.19]; 50 to < 70 years, IR = 5.23 [95% CI: 4.88, 5.58]; ≥ 70 years, IR = 10.06 [95% CI: 9.44, 10.68]) was observed with increased age (Supplementary Fig. 2). Based on 3,182 events of myocardial infarction, 2,814 events of stroke, 5,890 MACE, and 1,864 VTEs observed in the first 30, 45, 60, or 90 days after herpes zoster, there was no significantly increased risk for myocardial infarction, stroke, or MACE (Table 3). The IRR ranges (min–max) for each outcome for time intervals up to 90 days after herpes zoster were as follows: myocardial infarction (IRR = 1.31–1.81), stroke (IRR = 0.95–1.36), MACE (IRR = 0.93–1.23), and VTE (IRR = 1.58–2.13) (Table 4; Fig. 3). An increased risk of VTE was observed in the first 60 days (IRR = 2.13 [95% CI: 1.13, 4.02]) and 90 days (IRR = 2.13 [95% CI: 1.22, 3.72]) after herpes zoster. Additionally, when outcomes were stratified by age an increased risk of myocardial infarction (< 50 years, IRR = 1.78 [95% CI: 1.45, 2.12]; 50 to < 70 years, IRR = 8.43 [95% CI: 7.98, 8.87]; ≥ 70 years, IRR = 18.94 [95% CI: 18.09, 19.79]), stroke (< 50 years, IRR = 1.78 (1.45, 2.11); 50 to < 70 years, IRR = 6.57 (6.17, 6.97); ≥ 70 years, IRR = 18.10 [95% CI: 17.26, 18.93]), MACE (< 50 years, IRR = 3.72 [95% CI: 3.24, 4.20]; 50 to < 70 years, IRR = 14.92 [95% CI: 14.32, 15.52]; ≥ 70 years, IRR = 37.07 [95% CI: 35.86, 38.28]), and VTE (< 50 years, IRR = 1.85 [95% CI: 1.51, 2.19]; 50 to < 70 years, IRR = 5.23 [95% CI: 4.88, 5.58]; ≥ 70 years, IRR = 10.06 [95% CI: 9.44, 10.68]) was observed with increased age (Supplementary Fig. 2). Supplementary Fig. 3 describes the referent periods and risk intervals for cardiovascular events.

**Table 4:** Relative risk of cardiovascular outcomes following herpes zoster (time after infection)

	**IRR (95% CI) of outcome**			
**Time interval after first incident of herpes zoster**	**Myocardial infarction**	**Stroke**	**MACE**	**VTE**
30 days	1.81 (0.91, 3.63)	0.95 (0.35, 2.62)	1.23 (0.68, 2.23)	1.60 (0.63, 4.05)
45 days	1.66 (0.90, 3.06)	0.97 (0.42, 2.25)	1.13 (0.67, 1.89)	1.58 (0.71, 3.52)
60 days	1.48 (0.84, 2.64)	1.36 (0.72, 2.59)	1.19 (0.76, 1.87)	2.13 (1.13, 4.02)
90 days	1.31 (0.78, 2.20)	1.02 (0.55, 1.89)	0.93 (0.60, 1.42)	2.13 (1.22, 3.72)

*CI* confidence interval, *IRR* incidence rate ratio, *MACE* major adverse cardiovascular events, *VTE* venous thromboembolic event

## Discussion

There are limited data exploring the effectiveness and safety of Shingrix in patients with inflammatory musculoskeletal diseases who are receiving immunosuppressant medications. In this study, we described the characteristics of a population of patients with inflammatory arthritis (80% with rheumatoid arthritis) receiving Shingrix, a recombinant, adjuvanted VZV vaccine; assessed the effectiveness of Shingrix in this population; and estimated the risk of cardiovascular events following herpes zoster among these patients. The IR of herpes zoster after at least one dose of Shingrix was 7.41 per 1,000 PY compared with 14.76 per 1,000 PY without vaccination, yielding a crude vaccine effectiveness of 50% (95% CI: 44, 55), appreciably lower than that found in studies of older patients in the general population without immunosuppressive conditions [16, 29]. A post-marketing assessment of Shingrix vaccine effectiveness in Medicare beneficiaries aged > 65 years found a vaccine effectiveness of 64.1% in immunocompromised individuals and 68.0% in patients with autoimmune conditions, compared with 70.5% in the immunocompetent population [16]. A systematic review and meta-analysis that compared adjuvanted VZV in immunocompetent and immunocompromised individuals showed a vaccine effectiveness of 70% and 65% in immunocompetent and immunocompromised individuals, respectively [29]. Lower vaccine effectiveness of adjuvanted VZV in immunocompromised individuals may be explained by cell-mediated immunodeficiency and a weaker immune response in these individuals [29]. Disease-modifying antirheumatic drug (DMARD) therapy in patients with inflammatory musculoskeletal diseases can impact vaccine response. The effects of DMARDs on vaccines is variable; however, consistent themes emerge [30]. Rituximab substantially reduces antibody response, although T-cell responses may be preserved; methotrexate and abatacept reduce the immunogenicity of many vaccines; and tumor necrosis factor inhibitors and JAK inhibitors may reduce absolute post-vaccination antibody titers, although most patients still achieve seroprotection [30]. Of note, patients with inflammatory rheumatic diseases treated with JAK inhibitors exhibited lower seroconversion rates and T-cell responses compared with healthy controls [15], while phase 3 clinical trials of adjuvanted VZV have typically excluded patients on immunosuppressive therapy, limiting the generalizability of these findings [11, 30]. Vaccine effectiveness under field conditions depends on numerous factors, including type of vaccine, timing and age at vaccination, disease, immunocompromised status, sex, and the presence of comorbidities [11]. The 2022 ACR guideline for vaccinations in patients with rheumatic and musculoskeletal diseases includes guidance on whether to hold immunosuppressive medications or delay vaccination to maximize vaccine immunogenicity [12]; however, the decision to hold medication should consider the patient’s diagnosis, disease activity, risk of flare, and risk of vaccine-preventable infection, and should be a shared decision with the patient [12]. More studies are needed to investigate how effective Shingrix is in these patients and whether its effectiveness can be enhanced by short-term interruption of immunosuppressive medication or adjustment of vaccination timing.

Patients with rheumatoid arthritis and psoriatic arthritis have a higher risk of MACE than individuals without these conditions, and this could be, at least in part, due to the associated underlying chronic inflammation; however, the pathogenesis of cardiovascular disease in patients with inflammatory arthritis is complex and involves several intermediate factors [31–36]. In addition, patients with inflammatory arthritis are at a greater risk of herpes zoster. Current treatment guidelines for inflammatory arthritis recommend treatment with csDMARDs, bDMARDs, and targeted synthetic DMARDs (e.g., JAK inhibitors) [37–41]. Herpes zoster is more common in patients treated with JAK inhibitors than with csDMARDs or bDMARDs [32] and is associated with several serious chronic complications, such as post-herpetic neuralgia and herpes zoster ophthalmicus [35, 42, 43]. There is also growing evidence implicating VZV-related vasculopathy in the pathogenesis of cardiovascular disorders including stroke, coronary heart disease, myocardial infarction, and heart failure [44, 45]. Epidemiologic studies of herpes zoster and risk of myocardial infarction and stroke show an increased risk of these conditions close to the herpes zoster event; however, there is limited information on the long-term association between herpes zoster and cardiovascular events [44]. It has been reported that the risk of cardiovascular events and VTEs may be increased following herpes zoster [17–24]; thus, vaccination may indirectly reduce the risk of such events by reducing herpes zoster incidence. In this study, there was no significantly increased risk of myocardial infarction, stroke, or MACE observed in the first 30, 45, 60, or 90 days after herpes zoster, although numeric trends suggested an early increase in risk for myocardial infarction and VTE. However, there was a significantly increased risk of VTE observed in the first 60 and 90 days after herpes zoster (IRR = 2.13 for both). This is in line with the findings of a previous study in a large cohort of older patients (≥ 65 years old) with autoimmune inflammatory diseases, in which a significantly elevated and time-dependent risk for hospitalized stroke following herpes zoster, particularly in patients with cranial nerve involvement, was observed, and was greatest during the 0–90 days after herpes zoster [17].

Patients with rheumatic and musculoskeletal diseases are at higher risk of herpes zoster than older adults recommended for vaccination [12]. Recombinant VZV vaccination has been shown to be effective, with an acceptable safety profile in immunosuppressed patients undergoing renal transplantation, autologous stem cell transplantation, and in patients with hematologic malignancies, many of whom are < 50 years old. As such, the ACR recommends recombinant VZV vaccination in patients aged ≥ 18 years and < 50 years with rheumatic and musculoskeletal diseases who are taking immunosuppressive medication, as well as in the general population aged ≥ 50 years [12, 46–50].

Baseline demographics and patient characteristics stratified by Shingrix use indicated that the likelihood of patients receiving Shingrix vaccination increases with age, and also that patients with rheumatoid arthritis were more likely to be vaccinated than those with psoriatic arthritis or axial spondyloarthritis. Patients who were vaccinated were more likely to have dyslipidemia, hypertension, or ischemic heart disease compared with those who were not vaccinated. However, total uptake of the Shingrix vaccine in this population of patients with inflammatory arthritis was limited; only 21.6% of the total cohort received at least one dose of Shingrix. Despite CDC ACIP recommendations and ACR vaccination recommendations, encouraging vaccination in younger patients, only 3.7% of individuals < 50 years old were vaccinated with Shingrix in this study. Similarly, limited uptake has previously been observed for Zostavax [44, 51, 52]. A number of factors may have contributed to this low uptake of Zostavax, particularly that Zostavax was not recommended for use in patients on immunosuppressive therapies because it was a live attenuated vaccine and was therefore associated with a risk for herpes zoster infection in these patients [53]. However, this concern does not apply to Shingrix as a recombinant vaccine. Nevertheless, it may be that physicians and/or patients could have concerns over the safety of Shingrix in general [53, 54]. More specific to rheumatology patients, lack of understanding of herpes zoster prevalence and vaccination guidelines, underestimation of the real dangers of herpes zoster, along with insurance coverage and costs, may also have contributed to the low uptake [12, 54]. There may also be lack of assurance on its short- and long-term effectiveness in patients with inflammatory arthritis [54], concerns regarding flare of inflammatory arthritis given the potency of Shingrix and its mode of action [55], uncertainties about the need and best strategy for additional doses if efficacy wanes over extended periods, and the recommendation for patients to either pause some immunomodulatory medications or have well-controlled disease when they receive the vaccine [12], which may all limit uptake.

Limitations of this study include the fact that it was a retrospective, observational study of insurance claims using diagnostic codes for reimbursement purposes, and therefore only captured individuals who sought medical attention for herpes zoster and those who received Shingrix via their managed care organization. Therefore, the results may not be generalizable beyond the patients included in the database, which is a common limitation in claims data-driven studies. In addition, case algorithms for definition of cardiovascular outcomes can depend on the research question (e.g., broad vs. narrow definition, outpatient vs. inpatient, etc.), and their validity may not be consistent across studies. To increase specificity, our outcome definition of herpes zoster required concomitant antiviral use, but given the data source, polymerase chain reaction confirmation of herpes zoster was not feasible. We did not analyze complicated zoster (e.g., multidermatome, herpes zoster ophthalmicus), or other neurologic complications (e.g., cranial nerve involvement), which are known risk factors for further increased risk for cardiovascular events. Furthermore, we did not examine the immunomodulatory treatments being received at the time of vaccination, and it is possible that some therapies (e.g., JAK inhibitors, methotrexate, higher-dose glucocorticoid use) might have attenuated adjuvanted VZV effectiveness in those individuals. Finally, medication use was reported at any time on or after cohort entry, but the study did not examine the immunomodulatory treatments received at the time of vaccination, or adjust for immunomodulatory treatments received during follow-up as time-varying confounders, and it is possible that patterns of use may have differed between vaccinated and unvaccinated patients. However, this may not be a meaningful concern in that we do not have evidence to suggest that these therapies are effect modifiers that would attenuate relative vaccine effectiveness. The inability to adjust for all therapies in use may be addressed in future prospective studies.

## Conclusions

In summary, the data showed that the crude effectiveness of Shingrix in patients with inflammatory arthritis receiving typical immunomodulatory treatment was 50%. As the evidence points to the risk of VTE increasing in the 60–90 days after herpes zoster in patients with inflammatory arthritis, vaccination against herpes zoster may indirectly reduce the risk of such events in this already at-risk population by reducing herpes zoster incidence. Taken together, the findings of this study support the recommendation that adult patients with inflammatory arthritis should receive vaccination against herpes zoster. 

## Supplementary Information

Supplementary Material 1. Supplementary Table 1 ICD10 codes for inflammatory arthritis conditions. Supplementary Table 2 List of covariates. Supplementary Table 3 List of rheumatologic medications. Supplementary Table 4 Baseline demographics and patient characteristics stratified by Shingrix use. Supplementary Fig. 1 IR of herpes zoster stratified by age. Error bars represent 95% CI. CI confidence interval, IR incidence rate, PY person-years. Supplementary Fig. 2 Summary of key data stratified by age (i.e., relative incidence rates at ages < 50, ≥ 50 to <70, and ≥70 years). Supplementary Fig. 3 Referent periods and risk intervals for CV events.

## Data Availability

AbbVie is committed to responsible data sharing regarding the studies we sponsor. This includes access to anonymized, individual, and trial-level data (analysis datasets), as well as other information (e.g., protocols and clinical study reports), provided the studies are not part of an ongoing or planned regulatory submission. This includes requests for study data for unlicensed products and indications. These study data can be requested by any qualified researchers who engage in rigorous, independent scientific research, and will be provided following review and approval of a research proposal and statistical analysis plan, and execution of a Data Sharing Agreement. Data requests can be submitted at any time and the data will be accessible for 12 months, with possible extensions considered. For more information on the process, or to submit a request, visit https://www.abbvieclinicaltrials.com/hcp/data-sharing/.

## Abbreviations

ACR: American College of Rheumatology
AIDS: Acquired immune deficiency syndrome
axSpA: Axial spondyloarthritis
bDMARD: Biologic disease-modifying antirheumatic drug
CDC ACIP: Centers for Disease Control Advisory Committee on Immunization Practices
CI: Confidence interval
csDMARD: Conventional synthetic disease-modifying antirheumatic drug
DMARD: Disease-modifying antirheumatic drug
ER: Emergency room
FDA: Food and Drug Administration
HIV: Human immunodeficiency virus
IR: Incidence rate
IRR: Incidence rate ratio
JAK: Janus kinase
MACE: Major adverse cardiovascular events
NSAID: Non-steroidal anti-inflammatory drug
Optum CDM: Optum™ Clinformatics™ Data Mart
PsA: Psoriatic arthritis
PY: Person-years
RA: Rheumatoid arthritis
SCCS: Self-controlled case series study
SD: Standard deviation
VTE: Venous thromboembolic event
VZV: Varicella zoster virus
