# Supplementary material for: Real-world data on the use of the Shingrix vaccine among patients with inflammatory arthritis and risk of cardiovascular events following herpes zoster
Source: Arthritis Res Ther. 2025 May 17;27:108. doi: 10.1186/s13075-025-03565-0 (PMC12085024; doi:10.1186/s13075-025-03565-0)
Supplement: Supplementary file 1 — Supplementary Material 1. Supplementary Table 1 ICD10 codes for inflammatory arthritis conditions. Supplementary Table 2 List of covariates. Supplementary Table 3 List of rheumatologic medications. Supplementary Table 4 Baseline demographics and patient characteristics stratified by Shingrix use. Supplementary Fig. 1 IR of herpes zoster stratified by age. Error bars represent 95% CI. CI confidence interval, IR incidence rate, PY person-years. Supplementary Fig. 2 Summary of key data stratified by age (i.e., relative incidence rates at ages < 50, ≥ 50 to <70, and ≥70 years). Supplementary Fig. 3 Referent periods and risk intervals for CV events. [file 13075_2025_3565_MOESM1_ESM.docx]

**Supplementary Materials**

**Supplementary Table 1** ICD10 codes for inflammatory arthritis conditions

| **Diagnosis** | **ICD-10 codes** |
| --- | --- |
| **Rheumatoid arthritis** | M05, M05.0, M05.00, M05.01, M05.011, M05.012, M05.019, M05.02, M05.021, M05.022, M05.029, M05.03, M05.031, M05.032, M05.039, M05.04, M05.041, M05.042, M05.049, M05.05, M05.051, M05.052, M05.059, M05.06, M05.061, M05.062, M05.069, M05.07, M05.071, M05.072, M05.079, M05.09, M05.1, M05.10, M05.11, M05.111, M05.112, M05.119, M05.12, M05.121, M05.122, M05.129, M05.13, M05.131, M05.132, M05.139, M05.14, M05.141, M05.142, M05.149, M05.15, M05.151, M05.152, M05.159, M05.16, M05.161, M05.162, M05.169, M05.17, M05.171, M05.172, M05.179, M05.19, M05.2, M05.20, M05.21, M05.211, M05.212, M05.219, M05.22, M05.221, M05.222, M05.229, M05.23, M05.231, M05.232, M05.239, M05.24, M05.241, M05.242, M05.249, M05.25, M05.251, M05.252, M05.259, M05.26, M05.261, M05.262, M05.269, M05.27, M05.271, M05.272, M05.279, M05.29, M05.3, M05.30, M05.31, M05.311, M05.312, M05.319, M05.32, M05.321, M05.322, M05.329, M05.33, M05.331, M05.332, M05.339, M05.34, M05.341, M05.342, M05.349, M05.35, M05.351, M05.352, M05.359, M05.36, M05.361, M05.362, M05.369, M05.37, M05.371, M05.372, M05.379, M05.39, M05.4, M05.40, M05.41, M05.411, M05.412, M05.419, M05.42, M05.421, M05.422, M05.429, M05.43, M05.431, M05.432, M05.439, M05.44, M05.441, M05.442, M05.449, M05.45, M05.451, M05.452, M05.459, M05.46, M05.461, M05.462, M05.469, M05.47, M05.471, M05.472, M05.479, M05.49, M05.5, M05.50, M05.51, M05.511, M05.512, M05.519, M05.52, M05.521, M05.522, M05.529, M05.53, M05.531, M05.532, M05.539, M05.54, M05.541, M05.542, M05.549, M05.55, M05.551, M05.552, M05.559, M05.56, M05.561, M05.562, M05.569, M05.57, M05.571, M05.572, M05.579, M05.59, M05.6, M05.60, M05.61, M05.611, M05.612, M05.619, M05.62, M05.621, M05.622, M05.629, M05.63, M05.631, M05.632, M05.639, M05.64, M05.641, M05.642, M05.649, M05.65, M05.651, M05.652, M05.659, M05.66, M05.661, M05.662, M05.669, M05.67, M05.671, M05.672, M05.679, M05.69, M05.7, M05.70, M05.71, M05.711, M05.712, M05.719, M05.72, M05.721, M05.722, M05.729, M05.73, M05.731, M05.732, M05.739, M05.74, M05.741, M05.742, M05.749, M05.75, M05.751, M05.752, M05.759, M05.76, M05.761, M05.762, M05.769, M05.77, M05.771, M05.772, M05.779, M05.79, M05.8, M05.80, M05.81, M05.811, M05.812, M05.819, M05.82, M05.821, M05.822, M05.829, M05.83, M05.831, M05.832, M05.839, M05.84, M05.841, M05.842, M05.849, M05.85, M05.851, M05.852, M05.859, M05.86, M05.861, M05.862, M05.869, M05.87, M05.871, M05.872, M05.879, M05.89, M05.9, M06, M06.0, M06.00, M06.01, M06.011, M06.012, M06.019, M06.02, M06.021, M06.022, M06.029, M06.03, M06.031, M06.032, M06.039, M06.04, M06.041, M06.042, M06.049, M06.05, M06.051, M06.052, M06.059, M06.06, M06.061, M06.062, M06.069, M06.07, M06.071, M06.072, M06.079, M06.08, M06.09, M06.1, M06.2, M06.20, M06.21, M06.211, M06.212, M06.219, M06.22, M06.221, M06.222, M06.229, M06.23, M06.231, M06.232, M06.239, M06.24, M06.241, M06.242, M06.249, M06.25, M06.251, M06.252, M06.259, M06.26, M06.261, M06.262, M06.269, M06.27, M06.271, M06.272, M06.279, M06.28, M06.29, M06.3, M06.30, M06.31, M06.311, M06.312, M06.319, M06.32, M06.321, M06.322, M06.329, M06.33, M06.331, M06.332, M06.339, M06.34, M06.341, M06.342, M06.349, M06.35, M06.351, M06.352, M06.359, M06.36, M06.361, M06.362, M06.369, M06.37, M06.371, M06.372, M06.379, M06.38, M06.39, M06.4, M06.8, M06.80, M06.81, M06.811, M06.812, M06.819, M06.82, M06.821, M06.822, M06.829, M06.83, M06.831, M06.832, M06.839, M06.84, M06.841, M06.842, M06.849, M06.85, M06.851, M06.852, M06.859, M06.86, M06.861, M06.862, M06.869, M06.87, M06.871, M06.872, M06.879, M06.88, M06.89, M06.9 |
| **Psoriatic arthritis** | L40.5, L40.50, L40.51, L40.52, L40.53, L40.54, L40.59 |
| **Ankylosing spondylitis** | M45.X (M45, M45.0, M45.1, M45.2, M45.3, M45.4, M45.5, M45.6, M45.7, M45.8, M45.9)  M45.AX (M45.A, M45.A0, M45.A1, M45.A2, M45.A3, M45.A4, M45.A5, M45.A6, M45.A7, M45.A8, M45.AB)  M46.8X (M46.8, M46.80, M46.81, M46.82, M46.83, M46.84, M46.85, M46.86, M46.87, M46.88, M46.89) |

Definitions obtained from the Cross-Immunology Analysis

NUCC Taxonomy Codes for Rheumatologists: 207RR0500X, 2080P0216X

*ICD* International Classification of Disease

**Supplementary Table 2** List of covariates

| **Baseline patient characteristics** | Age |
| --- | --- |
|  | Sex (male, female) |
|  | Race (White, Asian, Black, Hispanic, unknown) |
|  | Geographic region (USA: Northeast, South, Midwest, West; Puerto Rico; missing; unknown) |
| **Cardiovascular risk factors** | AIDS |
|  | Asthma |
|  | Alcohol use |
|  | Arrythmias (including atrial fibrillation, atrial flutter, ventricular fibrillation, and ventricular flutter) |
|  |  |
|  | Cancer |
|  | Chronic kidney disease |
|  | Chronic liver disease |
|  | Chronic obstructive pulmonary disease |
|  | Congestive heart failure |
|  | Dementia |
|  | Depression |
|  | Diabetes |
|  | Dyslipidemia |
|  | Family history of ischemic heart disease |
|  | Hemiplegia and paraplegia |
|  | Herpes simplex virus |
|  | History of drug abuse |
|  | Hypertension |
|  | Ischemic heart disease |
|  | Liver disease |
|  | Myocardial infarction |
|  | Obesity |
|  | Peptic ulcer disease |
|  | Peripheral vascular disease |
|  | Renal disease |
|  | Rheumatologic disease |
|  | Smoking |
|  | Stroke |
| **Baseline medication use** | Opioids |
|  | NSAIDs |
| **Baseline healthcare utilization** | Number of rheumatologist visits |
|  | Number of outpatient visits (including rheumatologist visits) |
|  | Number of inpatient hospitalizations |
|  | Number of emergency department visits |
| **Herpes zoster risk factors** | Prior Zostavax vaccination |

*AIDS* acquired immune deficiency syndrome, *NSAID* non-steroidal anti-inflammatory drug

**Supplementary Table 3** List of rheumatologic medications

| **csDMARDs** | Methotrexate |
| --- | --- |
|  | Leflunomide |
|  | Sulfasalazine |
|  | Hydroxychloroquine |
|  | Chloroquine |
|  | Apremilast |
| **bDMARDs** | Etanercept |
|  | Adalimumab |
|  | Infliximab |
|  | Certolizumab pegol |
|  | Golimumab |
|  | Abatacept |
|  | Rituximab |
|  | Tocilizumab |
|  | Sarilumab |
|  | Anakinra |
| **JAK inhibitors** | Tofacitinib |
|  | Baricitinib |
|  | Upadacitinib |

*bDMARD* biologic disease-modifying antirheumatic drug, *csDMARD* conventional synthetic disease-modifying antirheumatic drug, *JAK* Janus kinase

**Supplementary Table 4** Baseline demographics and patient characteristics stratified by Shingrix use

|  | No Shingrix | ≥ 1 dose of Shingrix | ≥ 2 doses of Shingrix | Difference (Any – None) |
| --- | --- | --- | --- | --- |
| *N* | 103,982 | 28,690 | 21,012 |  |
| Age, mean (SD) | 58.90  (14.76) | 65.64  (9.29) | 66.13  (9.10) | 6.74  (6.60, 6.88) |
| Age groups, *n* (%) |  |  |  |  |
| < 50 | 27,479  (26.4) | 1,062  (3.7) | 620  (3.0) | -22.7  (-23.0, -22.4) |
| 50 to < 70 | 49,744  (47.8) | 17,551  (61.2) | 12,644  (60.2) | 13.4  (12.8, 14.0) |
| ≥ 70 | 26,759  (25.7) | 10,077  (35.1) | 7,748  (36.9) | 9.4  (8.8, 10.0) |
| Sex, *n* (%) |  |  |  |  |
| Male | 29,302  (28.2) | 7,915  (27.6) | 5,814  (27.7) | -0.6  (-1.2, 0.0) |
| Female | 74,680  (71.8) | 20,775  (72.4) | 15,198  (72.3) | 0.6  (0.0, 1.2) |
| Race, *n* (%) |  |  |  |  |
| White | 71,787  (69.0) | 21,061  (73.4) | 15,649  (74.5) | 4.4  (3.8, 5.0) |
| Asian | 2,888  (2.8) | 821  (2.9) | 606  (2.9) | 0.1  (-0.1, 0.3) |
| Black | 11,521  (11.1) | 2,842  (9.9) | 2,027  (9.6) | -1.2  (-1.6, 0.8) |
| Hispanic | 12,319  (11.8) | 2,790  (9.7) | 1,910  (9.1) | -2.1  (-2.5, 1.7) |
| Missing | 5,467  (5.3) | 1,176  (4.1) | 820  (3.9) | -1.2  (-1.5, 0.9) |
| Geographic region, *n* (%) | | | | |
| Northeast | 11,460  (11.0) | 3,094  (10.8) | 2,273  (10.8) | -0.2  (-0.6, 0.2) |
| South | 51,909  (49.9) | 12,835  (44.7) | 9,178  (43.7) | -5.2  (-5.9, 4.5) |
| Midwest | 24,537  (23.6) | 7,398  (25.8) | 5,581  (26.6) | 2.2  (1.6, 2.8) |
| West | 16,004  (15.4) | 5,351  (18.7) | 3,974  (18.9) | 3.3  (2.8, 3.8) |
| Missing/unknown/  Puerto Rico | 72  (0.1) | 12  (0.0) | 6  (0.0) | -0.1  (-0.1, 0.1) |
| Inflammatory arthritis diagnosis, *n* (%) | | | | |
| RA | 82,616  (79.5) | 23,482  (81.8) | 17,265  (82.2) | 2.3  (1.8, 2.8) |
| PsA | 17,373  (16.7) | 4,498  (15.7) | 3,252  (15.5) | -1.0  (-1.5, 0.5) |
| axSpA | 5,591  (5.4) | 1,052  (3.7) | 743  (3.5) | -1.7  (-2.0, 1.4) |
| Comorbidities, *n* (%) |  |  |  |  |
| Alcohol use | 2,692 (2.6) | 806 (2.8) | 591 (2.8) | 0.2 (0.0, 0.4) |
| Asthma | 18,863 (18.1) | 5,899 (20.6) | 4,286 (20.4) | 2.5 (2.0, 3.0) |
| Arrhythmias | 6,812 (6.6) | 2,192 (7.6) | 1,667 (7.9) | 1.0 (0.7, 1.3) |
| Chronic kidney disease | 11,736 (11.3) | 4,034 (14.1) | 2,975 (14.2) | 2.8 (2.4, 3.2) |
| Chronic liver disease | 15,835 (15.2) | 4,920 (17.1) | 3,613 (17.2) | 1.9 (1.4, 2.4) |
| Chronic obstructive   pulmonary disease | 23,100 (22.2) | 7,005  (24.4) | 5,101  (24.3) | 2.2  (1.6, 2.8) |
| Congestive heart failure | 8,563  (8.2) | 2,352  (8.2) | 1,686  (8.0) | 0.0  (-0.4, 0.4) |
| Depression | 27,880 (26.8) | 8,224  (28.7) | 5,863  (27.9) | 1.9  (1.3, 2.5) |
| Diabetes | 27,110 (26.1) | 8,538  (29.8) | 6,180  (29.4) | 3.7  (3.1, 4.3) |
| Drug abuse | 6,163  (5.9) | 1,605  (5.6) | 1,123  (5.3) | -0.3  (-0.6, 0.0) |
| Dyslipidemia | 59,037  (56.8) | 20,215  (70.5) | 14,981  (71.3) | 13.7  (13.1, 14.3) |
| Herpes simplex virus | 3,210  (3.1) | 1,112  (3.9) | 846  (4.0) | 0.8  (0.6, 1.0) |
| Herpes zoster   (plus antivirals) | 3,894  (3.7) | 1,502  (5.2) | 1,116  (5.3) | 1.5  (1.2, 1.8) |
| Hypertension | 63,708  (61.3) | 20,533  (71.6) | 15,083  (71.8) | 10.3  (9.7, 10.9) |
| Ischemic heart disease | 19,877  (19.1) | 6,654  (23.2) | 4,930  (23.5) | 4.1  (3.6, 4.6) |
| Family history of   ischemic heart disease | 8,191  (7.9) | 2,750  (9.6) | 2,039  (9.7) | 1.7  (1.3, 2.1) |
| Myocardial infarction | 1,678 (1.6) | 462 (1.6) | 346 (1.6) | 0.0  (-0.2, 0.2) |
| Stroke | 1,471  (1.4) | 407  (1.4) | 294  (1.4) | 0.0  (-0.2, 0.2) |
| MACE | 3,238  (3.1) | 911  (3.2) | 669  (3.2) | 0.1  (-0.1, 0.3) |
| VTE | 1,259  (1.2) | 357  (1.2) | 258  (1.2) | 0.0  (-0.1, 0.1) |
| Obesity | 38,288 (36.8) | 11,181 (39.0) | 8,016  (38.1) | 2.2  (1.6, 2.8) |
| Peripheral vascular   disease | 16,222 (15.6) | 5,419  (18.9) | 4,003  (19.1) | 3.3  (2.8, 3.8) |
| Smoking (ever) | 32,010 (30.8) | 9,315  (32.5) | 6,863  (32.7) | 1.7  (1.1, 2.3) |
| Current smoking   (past year) | 5,723  (5.5) | 1,207  (4.2) | 804  (3.8) | -1.3  (-1.6, -1.0) |
| Medications, *n* (%) |  |  |  |  |
| Opioid use | 60,712 (58.4) | 18,221 (63.5) | 13,415 (63.8) | 5.1  (4.5, 5.7) |
| Zostavax vaccination | 3,562 (3.4) | 2,562 (8.9) | 2,039 (9.7) | 5.5 (5.2, 4.8) |
| Shingrix vaccination | 0  (0.0) | 8,518  (29.7) | 6,669  (31.7) | 29.7  (29.2, 30.2) |
| Healthcare utilization | | | | |
| ER visit (past year), *n* (%) | 26,115 (25.1) | 7,212  (25.1) | 5,166  (24.6) | 0.0  (-0.6, 0.6) |
| ER visit (ever), *n* (%) | 45,600 (43.9) | 13,676 (47.7) | 10,092 (48.0) | 3.8  (3.1, 4.5) |
| Outpatient   rheumatologist visit   (past year), *n* (SD) | 2.88  (2.12) | 2.86  (2.00) | 2.86  (2.01) | -0.02  (-0.05, 0.01) |
| Outpatient   rheumatologist visit   (ever), *n* (SD) | 7.56  (10.46) | 8.30  (11.00) | 8.53  (11.25) | 0.74  (0.60, 0.88) |
| Outpatient visit  (past year), *n* (SD) | 10.74  (7.70) | 11.80  (8.02) | 11.81  (8.10) | 1.06  (0.96, 1.16) |
| Outpatient visit  (ever), *n* (SD) | 36.43  (41.63) | 44.93  (50.75) | 46.46  (52.81) | 8.50  (7.86, 9.14) |

Covariate status was assessed across all available data before cohort entry unless otherwise stated.

*axSpA* axial spondyloarthritis, *ER* emergency room, *MACE* major adverse cardiovascular events, *PsA* psoriatic arthritis, *RA* rheumatoid arthritis, *SD* standard deviation, *VTE* venous thromboembolic event


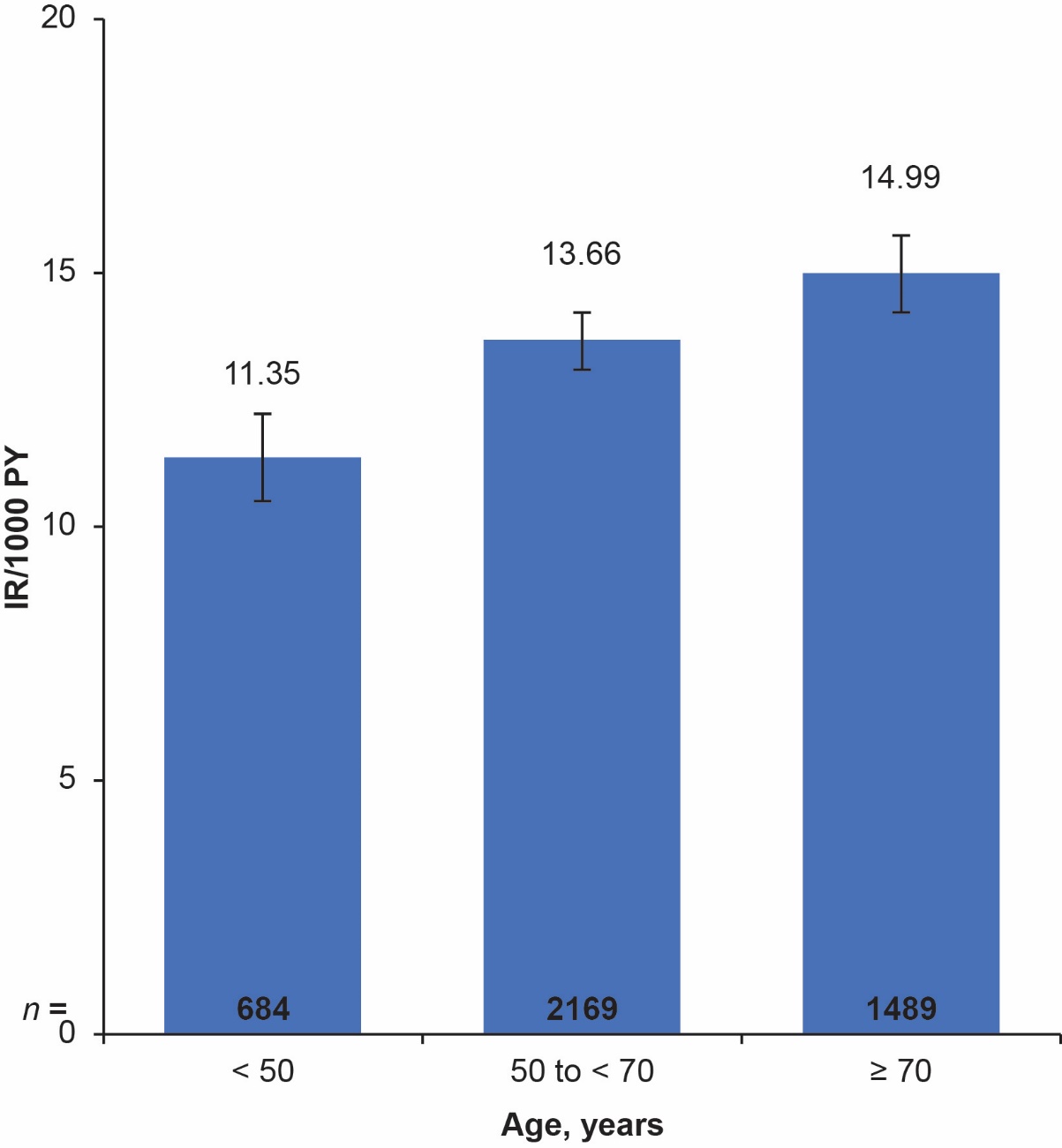


**Supplementary** **Fig. 1** IR of herpes zoster stratified by age. Error bars represent 95% CI.
*CI* confidence interval, *IR* incidence rate, *PY* person-years


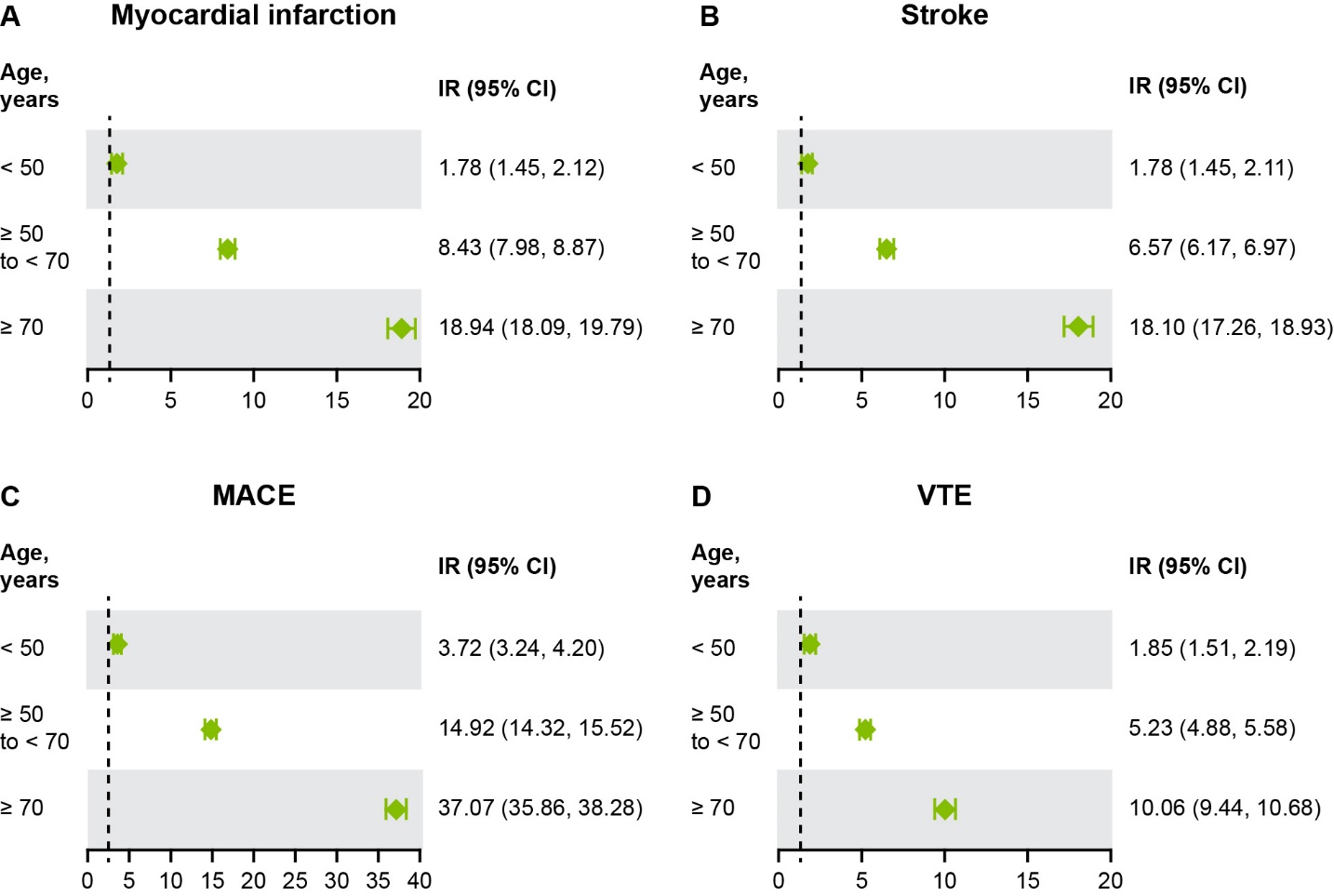


**Supplementary Fig. 2** Summary of key data stratified by age (i.e., relative incidence rates at ages < 50, ≥ 50 to <70, and ≥70 years)

*CI* confidence interval, *IR*  incidence rate, *MACE* major adverse cardiovascular events, *VTE* venous thromboembolic event


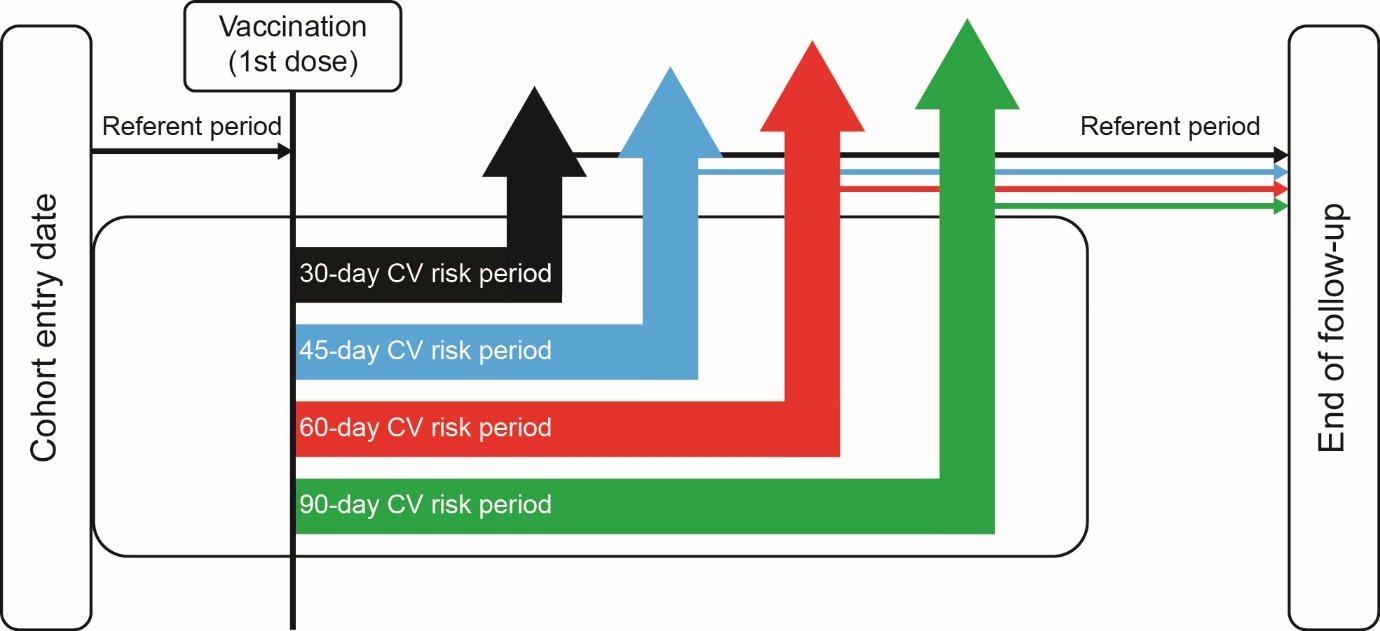


**Supplementary Fig. 3** Referent periods and risk intervals for CV events

*CV* cardiovascular
